# Synthesis of Metabolites and Metabolite-like Compounds Using Biocatalytic Systems

**DOI:** 10.3390/metabo13101097

**Published:** 2023-10-19

**Authors:** Roland Wohlgemuth

**Affiliations:** 1 MITR, Institute of Applied Radiation Chemistry, Faculty of Chemistry, Lodz University of Technology, Zeromskiego Street 116, 90-924 Lodz, Poland; roland.wohlgemuth.1@p.lodz.pl; 2 Swiss Coordination Committee Biotechnology (SKB), 8021 Zurich, Switzerland; 3 European Society of Applied Biocatalysis (ESAB), 1000 Brussels, Belgium

**Keywords:** metabolite synthesis, biocatalytic systems, product recovery, isotope-labelled metabolites, metabolite-like compounds

## Abstract

Methodologies for the synthesis and purification of metabolites, which have been developed following their discovery, analysis, and structural identification, have been involved in numerous life science milestones. The renewed focus on the small molecule domain of biological cells has also created an increasing awareness of the rising gap between the metabolites identified and the metabolites which have been prepared as pure compounds. The design and engineering of resource-efficient and straightforward synthetic methodologies for the production of the diverse and numerous metabolites and metabolite-like compounds have attracted much interest. The variety of metabolic pathways in biological cells provides a wonderful blueprint for designing simplified and resource-efficient synthetic routes to desired metabolites. Therefore, biocatalytic systems have become key enabling tools for the synthesis of an increasing number of metabolites, which can then be utilized as standards, enzyme substrates, inhibitors, or other products, or for the discovery of novel biological functions.

## 1. Introduction

Traditional knowledge of bioresources for microbial, plant and animal metabolites, as well as their processing and application, has been contributing tremendously to quality of life for thousands of years. Small molecular weight natural products have accompanied humankind and supported their quality of life in highly relevant areas, such as nutrition, diagnostics and therapy of diseases, dyes, cosmetics, and well-being, with an ever-increasing knowledge base. Numerous achievements have been made in terms of the isolation and purification of metabolites from natural bioresources and the elucidation of their molecular structures, demonstrating their large structural diversity. Renewed interest in the small molecule domain of biology [1] and the structures and functions of natural products and metabolites have brought the spotlight back onto metabolism [2]. With the great variety of small molecular weight compounds formed by the metabolism of biological cells, such as human and animal-derived metabolites, natural products from microbes and plants [3], as well as the large number of derivatives formed by the metabolism of biological cells from synthetic new molecular entities, a unified definition of ‘metabolite’ as a small molecular weight compound formed by the metabolism of a biological cell is used here. Manifold interactions between metabolites and other biomolecules, such as proteins, DNA, RNA, or other metabolites, within the same as well as between other biological cells, are of much fundamental interest for biological sensing, controlling and regulating processes at the genetic, epigenetic, transcriptomic, and proteomic levels. As precise experimental investigations of metabolites and their interactions with other biomolecules, such as their role as a substrate, inhibitor, or activator of an enzyme, are only possible by having available and pure metabolites, synthetic access is essential.

The concept of the metabolome [4], coined 25 years ago, has contributed to revitalizing interest in metabolites and metabolic pathways. Analyses of large numbers of metabolites have been facilitated by significant advances in powerful analytical methodologies with high information content [5,6,7], such as mass spectrometry (MS) [8] and nuclear magnetic resonance (NMR) [9], on which known and unknown metabolites can be based. A growing number of large databases are focusing on: (a) metabolite analysis using MS [8] or NMR [10]; (b) metabolites, metabolic pathways, natural products, and small molecules of biological interest [11,12,13]; and (c) species-specific metabolites and metabolic pathways (see Table 1 for a selection of species-specific metabolite databases) [14,15,16,17,18,19,20,21,22,23], providing fast access to information on the increasing number of identified metabolites and metabolic pathways.

The development of analytical methodologies for identifying biologically active metabolites and the interactions between metabolites and proteins and other biomolecules [24,25,26] offers great opportunities for delineating the molecular mechanisms of numerous biological processes. These include the activities of proteins and their modulation by activators, inhibitors, allosteric regulation, or post-translational modification; the sensing of metabolites by riboswitches or post-transcriptional modification of RNA; and controlling gene expression.

The use of isotopes for labelling small molecules continues to be essential for analytical methodologies, from measuring metabolite concentrations in biological matrices and determining metabolite fluxes in biological organisms to the discovery of metabolic pathways [27,28,29]. Biocatalytic systems have therefore been important for the synthesis of isotope-labelled metabolites, whether through whole cell systems or isolated enzymes [30,31]. Synthesis has also been key for proving the correct molecular structure [32] and for the production of larger amounts of the pure metabolite [33,34]. While synthetic organic chemistry provides a significant repertoire of well-established reactions, safety, and health, environment and sustainability aspects have become of increasing importance in the industrial manufacturing processes of metabolites [35]. Improving resource and energy efficiency, reducing risks and the extensive use of natural resources, and avoiding the use of toxic chemicals are major planetary issues for sustainable development. Catalytic reactions provide novel, green, and sustainable methodologies for powerful and resource-efficient synthetic chemistry. This has also been convincingly demonstrated by recent Nobel Prizes in chemistry which were awarded in 2018 to Frances Arnold for directed evolution of enzymes [36] and in 2021 to Benjamin List [37] and David McMillan [38] for the development of asymmetric organocatalysis. Fundamental and mutually beneficial inspirations can originate from the interface and interactions between biocatalysis and organocatalysis [39]. Metabolic pathways used by biological organisms to prepare valuable metabolites from the raw materials available in their environment have also been successfully utilized and developed into industrial bioprocesses for manufacturing of metabolites, metabolite-like compounds, non-natural chemical entities, and metabolites thereof [40,41,42]. The metabolic pathways, which start from highly functionalized biobased raw materials instead of hydrocarbons, also provide inspirations for biocatalytic defunctionalization reactions in transitioning towards raw materials from bioresources [43]. However, the desired use of biobased raw materials also needs to consider other goals, such as biodiversity, sustainability, and supply chain issues, if biologically endangered and rare biological species are required or the amount and quality of the biobased raw material is variable and subject to various environmental factors [44]. Therefore, the molecular and engineering fundamentals of how nature achieves the biosynthesis of metabolites using biocatalytic reactions in microbes, plants, animals, and humans have attracted much interest as a blueprint for optimized biocatalytic systems of metabolite production [45].

The purpose of this work is to provide an overview of design and engineering approaches for biocatalytic systems in metabolite production and their application in manufacturing processes. The significance of biocatalytic systems for metabolite production is connected with the general strategic advantages of using biocatalysis in synthesis, such as their high selectivity and shortened synthetic routes [46].

## 2. Design and Engineering of Biocatalytic Systems

Biocatalytic system design and engineering towards the synthesis of metabolites starts with route selection and includes the preparation of suitable biocatalysts and raw materials, reaction engineering, process integration, intensification, and scaling of the selected metabolite manufacturing processes [35]. The main aim of this work focuses on designing and engineering biocatalytic systems in order to produce metabolites. Great progress has been made in delineating natural and engineering synthetic metabolic pathways, advanced methodologies and tools for finding and applying biocatalysts, and last but not least product recovery and purification. These significant advances along a whole bioprocess and workflow have brought biocatalytic systems into a privileged position. Biocatalytic systems are being used for producing not only natural metabolites, but also metabolite-like compounds; metabolites derived from the transformation of new chemical entities by biological organisms and isotope-labelled metabolites.

**Figure 1:**
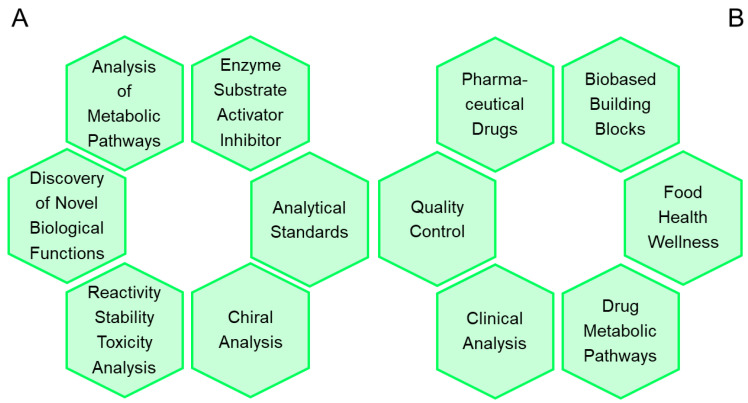
Selected reasons for the synthesis of metabolites and metabolite-like compounds. (**A**) For academic research applications in basic and applied sciences, where small quantities are usually needed, for example in biochemistry and other life sciences for the analysis of metabolic pathways, the discovery of unknown biological pathways, in preclinical research and development, and medical and diagnostic applications. (**B**) For applications in industry, where usually large quantities are needed of active ingredients and intermediates of pharmaceuticals, vitamins, flavors, fragrances, dyes, or agrochemicals. In clinical chemistry, standards are needed for blood and urine tests of biomarkers for diagnostic purposes and therapeutic drug monitoring or drug abuse. In medicinal chemistry, a diversity of test compounds and standards are needed for studying biological activity and efficacy, metabolism, and pharmacokinetics, and for determining the parameters of absorption, distribution, metabolism, excretion, and toxicity of novel molecular entities.

Having synthetic access to a metabolite or metabolite-like compound, or even more convenient, having an already available pure product, has been and continues to be highly important and relevant [47,48] for a number of reasons, as shown in Figure 1A for fundamental and applied sciences, as well as for a great variety of applications in industry and medicine, as shown in Figure 1B.

In view of millions of protein sequences, genomic enzymology tools are highly valuable for generating clues and hypotheses to guide experiments towards the correct assignment of enzyme and metabolic functions, as the functions of a significant fraction of protein sequences are unknown, uncertain, or even misassigned [49]. The final proof of the assignments is not possible without experimental verification of the predicted enzyme functions. This requires the availability of the corresponding metabolites (a) as enzyme substrates to demonstrate their conversion into the predicted product and to perform the enzyme activity assays, and (b) for the discovery of novel biological functions. In the case of chiral enzyme substrates or products, or for investigations of enzymatic reaction mechanisms, enantiopure metabolites can resolve fundamental questions and provide more detailed insights. Applications in industry are growing and include food supplements, pharmaceuticals, flavors, fragrances, cosmetics, dyes, and agrochemicals. The excellent chemo-, regio-, and stereoselectivity and mild reaction conditions of biocatalytic systems, which avoid protection–deprotection schemes, are environment friendly and safe to use. This is advantageous for the use of reactions requiring toxic chemicals, heavy metals, or the introduction and removal of protecting groups in chemocatalytic or stoichiometric reactions.

There are also disadvantages to overcome for biocatalytic systems when substrate and product concentrations are limited by solubility in aqueous media or enzyme inhibition. In these cases, a reaction type corresponding to a well-established key functionalization reaction in organic chemistry requires intense elaboration of a suitable biocatalytic systems for a selected substrate to product conversion due to the narrow substrate scope of the biocatalyst. Stability issues, for biocatalysts as well as for substrates and products, need to be checked, and in case of fragile biologically active metabolites, suitable operating windows for the reaction and workup must be selected. Improving energy and resource efficiency in manufacturing metabolites, such as reducing energy use and avoiding protection–deprotection schemes in lengthy chemical synthesis from fossil-based raw materials or minimizing biological waste in low-yield extractive procedures from biobased raw materials, is essential in building more resilient and sustainable manufacturing routes to metabolites. A resilient manufacturing route to a metabolite means a route with a robust and stable production process with the ability to effectively cope with changing boundary conditions, respond, and maintain reliable manufacturing. The molecular transformations catalyzed by enzymes and metabolic pathways in nature, as well as the great advances of biocatalysis, provide a blueprint and rich sources of knowledge for designing and engineering biocatalytic systems for the synthesis of metabolites.

**Figure 2:**
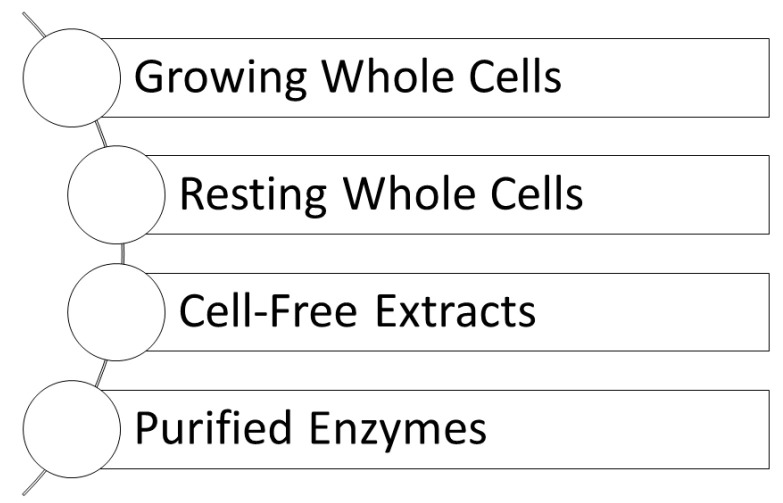
Basic types of biocatalytic systems for the synthesis of metabolites and metabolite-like compounds.

Biocatalytic systems using whole cells have been developed into numerous fermentation processes as well as biotransformation processes at large industrial scales for the production of metabolites [50,51,52,53]. Fermentation processes use the cultivation of microbial whole cells in a fermenter containing the medium with the nutrients required for cell growth and product biosynthesis, followed by subsequent product recovery and purification, while biotransformation processes make use of microbial whole cells in a resting state for transforming an advanced intermediate to the product under physiological conditions. The vast knowledge base and the rich diversity of biocatalytic whole-cell systems have led to biocatalytic metabolite production through suitable growing or resting whole cells (see Figure 2). Metabolic engineering and synthetic biology enable improvements in titer, rate, and space–time yield of metabolite synthesis from the starting materials (a) by increasing the performance of the biosynthetic pathways to the metabolite, and (b) by deleting any biocatalytic degradation reaction of the final metabolite and of any metabolic intermediates. Biocatalytic whole-cell systems are also connected with a high degree of complexity, which can be reduced by using cell-free biocatalytic systems (see Figure 2) in different forms of purification, from crude cell-free extracts to isolated and purified enzymes. Whatever biocatalytic system is considered, bioprocess design and engineering need to address and optimize various parameters such as biocatalytic pathway selection, form and status of the biocatalysts, reaction engineering, downstream processing, and purification of the metabolites [35,54]. Cell-free biocatalytic systems have the advantage of reducing the bioprocess complexity through the absence of interfering and degrading enzymes or the removal of mass-transfer limitations for substrates and products [54].

While the step economy of a biosynthetic pathway is already considered in the design phase, possible improvements such as complexity reduction, intermediate purification steps, and the number of separate reactors needed are also taken into account by the degree of process integration involved in biocatalytic metabolite synthesis. Depending on how many reaction steps are needed for transforming the starting materials to the metabolites, one-step to multistep enzymatic reactions are developed, if possible in one pot. If all the enzymatic reactions of a biosynthetic pathway can be performed well, enzymatic total synthesis can be achieved [55].

## 3. Synthesis of Naturally Occurring Metabolites

**Figure 3:**
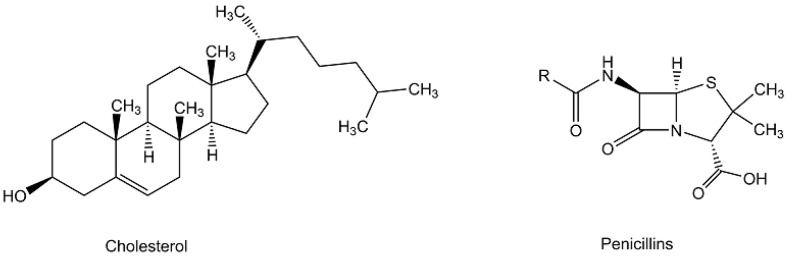
Selected naturally occurring metabolites with demonstrated total synthesis.

The milestone discoveries that small molecules of life could not only be isolated from nature, but could also be synthesized in the laboratory from inorganic chemical precursors, started the new era of synthetic organic chemistry [56]. The synthesis of urea by Friedrich Wöhler in 1828 [57] or acetic acid by Hermann Kolbe in 1845 [58] were clear demonstrations that organic compounds, which are found and formed naturally in living organisms, could be prepared starting from inorganic materials. This sparked tremendous interest in synthetic organic chemistry, and impressive advances have been achieved in the art and science of total synthesis of numerous more complex natural products (see Figure 3), such as cholesterol and penicillin [59,60,61,62,63]. Total synthesis has not only been key to the final proof of structures and to correcting mistakenly assigned structures [64], but it has also been a key driver for novel synthetic methods in organic chemistry, which remain at its heart up to the present time [65].

**Figure 4:**
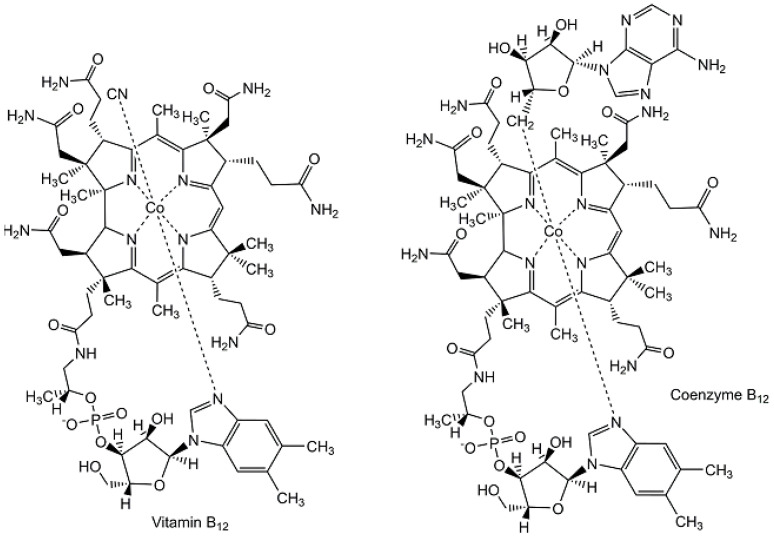
Structures of vitamin B_12_ and coenzyme B_12_.

The tools and methodologies used for broadly applicable synthetic reactions, either reactions which are already well established or newly discovered and emerging reactions from modern organic chemistry, have enabled the total synthesis of an impressive number of naturally occurring metabolites [66,67,68,69] to be synthesized from very simple structures without any stereogenic centers into the most complex metabolites (see Figure 3 and Figure 4). Synthesizing naturally occurring polycyclic metabolites, such as steroids, from simple building blocks has attracted much interest [66]. The first total synthesis of cholesterol was described by R.B. Woodward, whereby the methyl-3-ketoetio-*allo*-cholanate synthesized previously was converted via cholestan-3-ol, cholestane-3-one, and 4-cholesten-3-one into cholesterol [67,68]. With large amounts of industrially manufactured cholesterol extracted from animal-derived raw materials, new short and straightforward synthetic routes starting from non-animal-derived biobased raw materials are of much interest, such as the conversion of plant-based diosgenin to cholesterol in four steps [69]. Overcoming the great challenges of the chemical synthesis of vitamin B_12_, a microbial metabolite of high molecular complexity (see Figure 4), has not only resulted in many significant discoveries and novel methods on the road to that goal, but has led to the epochal milestone of its first total synthesis by the research groups of Alfred Eschenmoser in Zurich and R.B. Woodward in Cambridge [70,71]. The identification of the microbial enzymes and the aerobic and anaerobic enzymatic pathways to vitamin B_12_ in nature [72] have shown the power of biocatalytic total synthesis and are also of fundamental interest in the context of the origin of vitamin B_12_ and related compounds [73].

**Figure 5:**
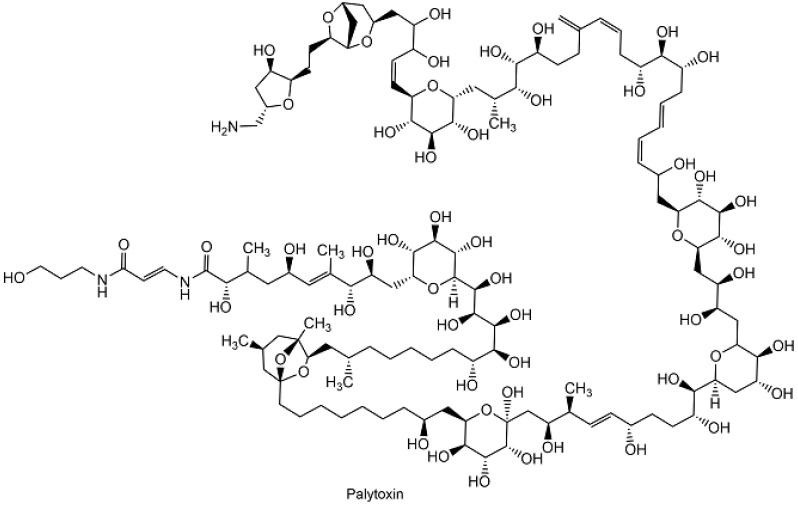
Complete stereochemistry and planar structure of palytoxin.

The tremendous task of establishing the complete stereochemistry and total synthesis of the marine natural product palytoxin (see Figure 5), which has a molar mass of 2680 and contains 63 stereogenic centers as well as four *trans*- and three *cis*-carbon-carbon double bonds, has been achieved by Yoshito Kishi and coworkers [74].

In this pioneering work and landmark achievement, eight building blocks were coupled in seven reactions to the fully protected palytoxin carboxylic acid containing 43 protecting groups in total, from which the palytoxin carboxylic acid was obtained in a 35% overall yield after removal of the protecting groups [74]. The palytoxin carboxylic acid was then treated with acetic acid in order to obtain the corresponding δ-lactone, which was then converted into the final palytoxin [74]. The development of new chemical reactions, such as the powerful Fe(III)-mediated coupling of catharanthine and vindoline to anhydrovinblastine, which may perhaps be also involved in the natural plant biosynthesis of vinblastine, has enabled the synthesis of vinblastine (see Figure 3) and related compounds in 8 to 13 reaction steps [75]. Total synthesis without using protecting groups is of significant interest for reducing the complexity, cost, and number of steps, as shown in the synthesis of marine natural products [76].

The milestone discoveries of living microbial whole cells [77], their biosynthetic capabilities, and the elucidation of the organic chemistry of the underlying biocatalytic reactions exerted by cell-free extracts [78] have started the era of synthetic biochemistry (see Table 2 for an overview of the natural metabolites covered in this review, with their respective pathways involved).

Although the biological formation of urea is known, the elucidation of urea biosynthesis in animals from ammonia and carbon dioxide [79,80] required major scientific breakthroughs. Early preparative applications of biocatalysts focused on particular reactions, such as stereoselective sugar oxidation through the use of microbial whole cells, stereoselective carbonyl reductions using baker’s yeast or alcohol dehydrogenases, kinetic resolutions, or desymmetrization reactions using hydrolases such as pig liver esterase (PLE).

**Figure 6:**
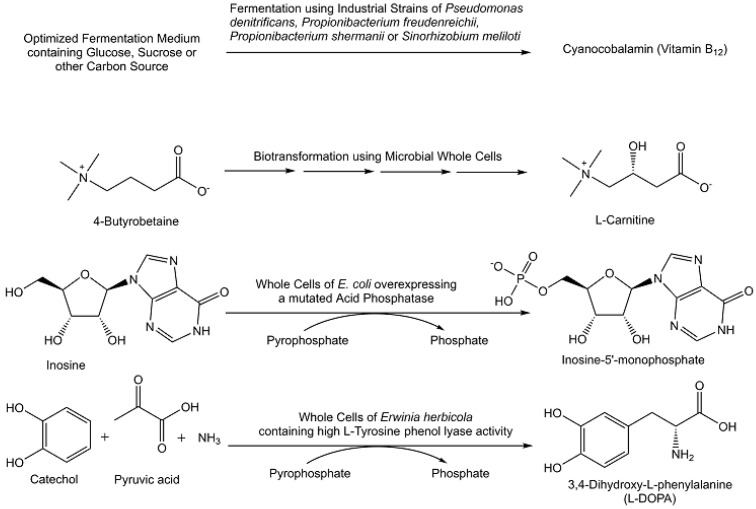
Selected naturally occurring metabolites produced through bioprocesses at an industrial large scale.

With the tremendous development of recombinant enzymes and enzyme engineering, a range of biocatalytic reaction platforms have become the first choice, such as asymmetric ketone reductions catalyzed by recombinant ketoreductases at a large scale. Numerous bioprocesses have been developed to an industrial large scale (see Figure 6) for producing naturally occurring metabolites, such as citric acid [81] and other organic acids, steroids [82], beta-lactams and other antibiotics [83], L-carnitin and other amino acids [84,85], vitamins [86], and microbial metabolites such as anticancer and immunosuppressant drugs [87]. Bioprocesses for functionalized and modified steroids, steroidal intermediates, and metabolites provide shortened routes compared with chemical synthesis and have been well established in the pharmaceutical industry for decades [88]. 17β-estradiol has been obtained with greater than 99% diastereomeric excess and a 64.8% yield through stereoselective reduction of estrone using *Saccharomyces cerevisiae* whole cells [89]. Industrial bioprocesses for the efficient production of 4-androstene-3,17-dione and related metabolites from phytosterols at high substrate concentrations [90] make these metabolites attractive precursors for the sustainable production of steroids from plant-based raw materials [82]. Therefore, the selective biocatalytic reduction of 4-androstene-3,17-dione to testosterone has attracted much interest among the various biocatalytic routes to testosterone. From an efficient and complete conversion of 4-androstene-3,17-dione at a substrate concentration of 28.8 g L^−1^, the product testosterone has been obtained with a purity of greater than 97% in 10 h [91].

Of particular interest for steroid bioprocesses are biocatalysts, which are able to catalyze highly selective reactions such as the regio- and stereoselective biocatalytic hydroxylation of unique C(sp^3^)-H positions of the steroid backbone [92], for example, the 17α-hydroxylase, the 21-hydroxylase, and the 11β-hydroxylase in the conversion of progesterone to hydrocortisone [93]. Bioprocesses for the biocatalytic synthesis of steroidal compounds from simple carbon sources are emerging [94], and the biocatalytic cyclization of linear precursors to the steroid backbone catalyzed by cyclases in one step is noteworthy [95]. Cyclases are also involved in the biocatalytic synthesis of all vitamin E components; α-, β-, γ-, and δ-tocopherol; and α-, β-, γ-, and δ-tocotrienol. Additionally, bioprocesses using in vitro plant cell cultures are of much interest [96] for the preparation of enantiomerically pure vitamin E components such as (*R*,*R*,*R*)-α-tocopherol. A new route to vitamin E has been established at a large scale by combining biocatalytic and chemical routes, such as the chemical synthesis of α-tocopherol via isophytol from β-farnesene, which is manufactured through fermentation [97]. Route shortening is also of much interest for the synthesis of lipid mediators, and a stereoselective biocatalytic Baeyer–Villiger oxidation and a diastereoselective ketoreductase-catalyzed enone reduction have been key steps in a chemoenzymatic synthesis strategy for prostaglandins [98].

**Figure 7:**
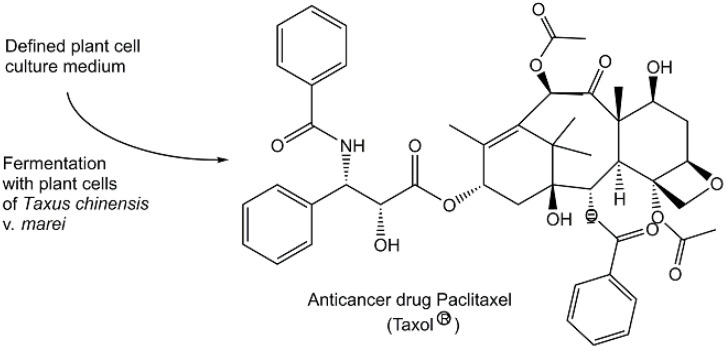
Plant cell fermentation for the industrial production of paclitaxel.

Natural metabolites continue to be highly important as sources for small molecule pharmaceuticals for treating diseases such as infectious diseases, cancer, cardiovascular diseases, diabetes, glaucoma, or multiple sclerosis [99], and large-scale manufacturing is key for adequate supply. Therefore, efficient and reliable bioprocesses for metabolites have been important to replace: (a) extraction processes from endangered biological species which may become extinct and which may give yields depending on various environmental factors, or (b) non-sustainable production procedures combining extraction from biological species and synthetic modifications. A sustainable plant cell fermentation process for producing the natural diterpenoid paclitaxel (registered trade name Taxol^(R)^) at an industrial large scale (see Figure 7) preserves *Taxus* plants and is able to provide the required amounts of this *Taxus* species metabolite, which the WHO lists as an essential medicine and is used in cancer treatment [100,101]. The cell culture medium contains a simple monosaccharide as a carbon source, one or more amino acids as a nitrogen source, and a silver ion or complex, jasmonic acid methyl ester, auxin-related growth regulators, and phenylpropanoid pathway inhibitors like 3,4-methylenedioxy-6-nitrocinnamic acid for enhancing the production of taxol [100]. Human health and the quality of life of a large number of people around the world have greatly benefitted from a long history of dedicated work to discover and develop small molecules, such as artemisinin and avermectins, formed by biological organisms as gifts from nature for their use as anti-infectives in the 20th century [102,103]. It is, however, not the time for complacency today, as the appearance of new infectious agents, human negligence, and economic boundary conditions require actions towards the discovery, development, and reliable production of novel anti-infectives [104,105,106].

**Figure 8:**
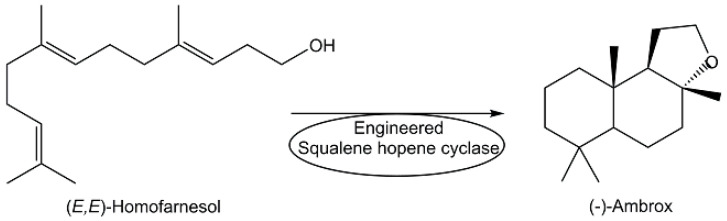
Enzymatic process for the industrial production of the fragrance ingredient (-)-Ambrox.

Beyond the manufacturing of metabolites through bioprocesses at industrial large scales such as anticancer drugs, anti-infectives, and other pharmaceuticals for human health [83,87], biomanufacturing of a variety of other metabolites such as vitamins [86,107], flavors, and fragrances [108] has become increasingly attractive for various industrial sectors [109]. Fermentation using *Pseudomonas denitrificans* or *Pseudomonas freudenreichii* strains downstream and purification processes has been developed for the manufacturing of vitamin B_12_ at an industrial large scale [109]. The efficient enzymatic cyclization of (*E*,*E*)-homofarnesol, which can be produced from the fermentation product (*E*)-β-farnesene, to the fragrance ingredient (-)-ambrox catalyzed by engineered squalene hopene cyclase at an industrial scale (see Figure 8) represents a significant improvement in carbon efficiency and sustainability [108].

Another important area is the synthesis of metabolites for analytical or diagnostic applications, such as the use of metabolites in analytical or diagnostic devices, as standards, or for measuring enzyme activities. The synthesis of metabolites which act as ionophores has been of much interest for the analysis and monitoring of biomedically and environmentally relevant ions via ion-selective electrodes and sensors [110], for example, the biomanufacturing of highly pure nonactin as a neutral ionophore in monitoring ammonium ions [111,112].

Metabolites also need to be synthesized for analytical investigations involving the measurement of enzyme activities, such as the analysis of enzyme activities relevant to clinical chemistry, food analysis, enzymology, enzyme production, environment, verification or discovery of novel enzyme functions, development of enzyme inhibitors, or the analysis of activating, signaling, or regulatory functions.

**Figure 9:**
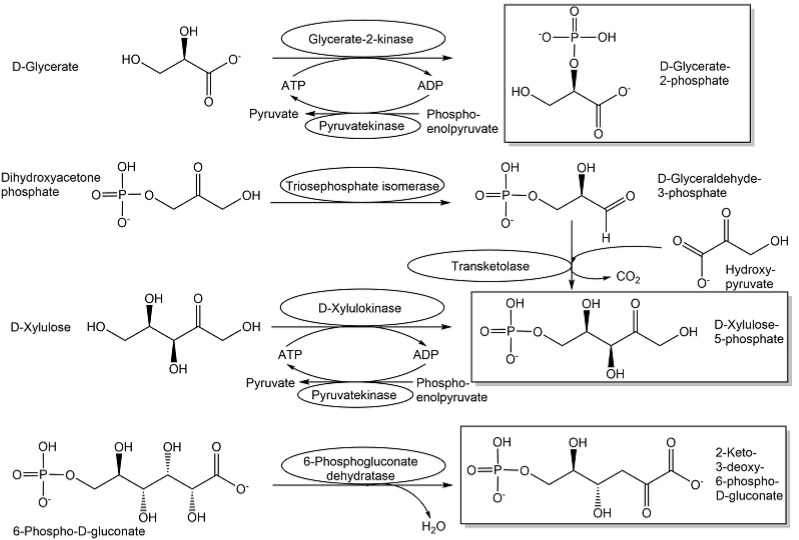
Biocatalytic synthesis of selected naturally occurring metabolites of the Emden–Meyerhof–Parnas, the Entner–Doudoroff, and the pentose phosphate pathways.

Energy metabolism and glycolytic pathways are central to biological organisms, and the metabolites of the monosaccharide catabolic pathways are essential. It is therefore desirable to synthesize, in pure and stable form, the metabolites (see Figure 9) of the most common pathways for the breakdown of D-glucose, the Emden–Meyerhof–Parnas, the Entner–Doudoroff, and the pentose phosphate pathway, because these are central to kingdoms of life.

The enantiomerically pure metabolite D-glyceraldehyde-3-phosphate, which occurs in all the three most common D-glucose catabolic pathways, as well as the enantiomerically pure L-glyceraldehyde-3-phosphate, which is toxic to cells, have been synthesized from their corresponding aldehydes through biocatalytic ATP-dependent phosphorylation using enantiocomplementary kinases and phosphoenol pyruvate/pyruvatekinase for regenerating ATP. Glycerol kinase has been used in the enantioselective phosphorylation of L-glyceraldehyde [113,114], which could be prepared through glycerol dehydrogenase-catalyzed resolution of racemic glyceraldehyde [115,116]. Dihydroxyacetone kinase has been found as the corresponding enantiocomplementary enzyme to enantioselectively catalyze the phosphorylation of D-glyceraldehyde [117]. The enolase substrate D-glycerate-2-phosphate has been prepared by phosphorylating D-glycerate using recombinant glycerate-2-kinase, ATP as cofactor, and phosphoenolpyruvate/pyruvatekinase for ATP regeneration [118]. In the pentose phosphate pathway, D-xylulose-5-phosphate has been prepared through two different routes; either through transketolase-catalyzed condensation of D-glyceraldehyde-3-phosphate [119,120] with hydroxypyruvate, which serves as irreversible C2-donor, or through xylulokinase-catalyzed ATP-dependent phosphorylation of D-xylulose [121,122]. This latter approach has also been extended to the synthesis of L-xylulose-5-phosphate by using an enantiocomplementary xylulokinase [121]. In the D-tagatose catabolic pathways, D-tagatose-6-phosphate 1-kinase-catalyzed phosphorylation of D-tagatose-6-phophate enabled the preparation of the central metabolite D-tagatose-1,6-diphosphate [122]. A characteristic metabolite for the Entner–Doudoroff pathway is 2-keto-3-deoxy-6-phosphogluconate, which can be synthesized in one step by eliminating water from 6-phosphogluconate using 6-phosphogluconate dehydratase [123]. In a similar way, metabolites of other monosaccharide non-phosphorylative catabolic pathways can be synthesized in a straightforward way from the corresponding sugar acid, such as 2-keto-3-deoxy-D-galactonate from D-galactonate or 2-keto-3-deoxy-D-xylonate from D-xylonate using D-xylonate dehydratase [124]. D-gluconate dehydratase-catalyzed water elimination from D-gluconate allows for the straightforward preparation of 2-keto-3-deoxy-D-gluconate [125]. In energy metabolism, the high energy of the phosphorus–nitrogen bond in phosphagens is a key energy source. Selective biocatalytic synthesis enables straightforward access, such as in the one step synthesis of *N*_ω_-phospho-L-arginine [126].

Biocatalytic methods are also very useful in synthesizing a number of key metabolites from other metabolic pathways. Shikimic acid-3-phosphate can be prepared through shikimate kinase-catalyzed ATP-dependent phosphorylation of shikimic acid and ATP regeneration using phosphoenolpyruvate and pyruvate kinase [127]. Pyridoxamine-5’-phosphate was synthesized from pyridoxal-5-phosphate through biocatalytic transamination using an ω-transaminase [128]. The biocatalytic L-arginine addition reaction to fumaric acid enabled efficient one-step access to the urea cycle metabolite L-argininosuccinate [129,130].

Biocatalytic methods have also been of much interest for the synthesis of vitamin D metabolites. Highly selective side-chain hydroxylation of vitamin D3 in the 25-position has been achieved at a laboratory scale using different biocatalytic approaches, such as cytochrome P450 monooxygenases, complex electron donors, and oxygen in whole-cell systems [131,132], through hydrogen peroxide-dependent peroxygenase [133], or through ferricyanide-dependent biocatalytic hydroxylation using a vitamin D3 hydroxylase as cell-free extract or as purified enzyme from *Sterolibacterium denitrificans* [134]. Efficient biocatalytic production of 573 mg of 25-hydroxyvitamin D3 per liter has been achieved using nisin-treated cells of *Rhodococcus erythropolis* containing an engineered vitamin D3 hydroxylase from *Pseudonocardia autotrophica* (573 mg of 25-hydroxyvitamin D3 per liter within 2 h) [131], and by using a *Bacillus cereus* strain (830 mg of 25-hydroxyvitamin D3 per liter within 60 h) [132]. A facile ferricyanide-dependent hydroxylation using a vitamin D3 hydroxylase, either as a cell-free extract or as purified enzyme from *Sterolibacterium denitrificans*, has enabled a simplified preparation of 25-hydroxyvitamin D3 in a yield greater than 99% at a 1 mM substrate concentration [134]. The biocatalytic synthesis of 1α,25-dihydroxyvitamin D3 (calcitriol) from vitamin D3 has been achieved through double hydroxylation of vitamin D3 using whole cells of *Pseudonocardia* sp., with more than 30% yield and a titer of approximately 62 mg L^−1^ [135]. Biocatalytic hydroxylation of 25-hydroxyvitamin D3 catalyzed by 25-hydroxyvitamin D3 24-hydroxylase and formation of 24*R*,25-dihydroxyvitamin D3 have been demonstrated on an analytical scale [136].

The 25-hydroxyvitamin D2 has been obtained as the sole product in a 90% yield through the regioselective hydroxylation of vitamin D2 catalyzed by a peroxygenase from *Coprinopsis cinerea* [133]. An engineered triple variant of CYP105A1 with increased 1α-hydroxylase activity has enabled the formation of 1α,25-dihydroxyvitamin D2, while an engineered double variant of CYP105A1 showed increased 26-hydroxylase activity and was best for the formation of 25,26-dihydroxyvitamin D2 [137]. Whole cells of *Bacillus megaterium* expressing the highly selective vitamin D2 hydroxylase CYP109E1 were used for the biocatalytic two-step hydroxylation of vitamin D2, whereby a titer of 12 mg L^−1^ of 24*R*,25-dihydroxyvitamin D2 was obtained in 48 h [138]. Simple biocatalytic routes are also of special interest for disease-specific metabolites in order to support and simplify the diagnostics, for example, of inborn errors of metabolism, cancers, and cardiovascular and metabolic diseases [139,140,141].

## 4. Synthesis of Isotope-Labelled Metabolites

The use of radioactive isotopes such as ^3^H (tritium), ^14^C, or ^32^P has been instrumental for the discovery of major metabolic pathways, such as the path of carbon in photosynthesis [142]. Biocatalytic methods, which have been developed for the synthesis of metabolites labelled with a radioactive isotope at a specific position, such as tritium- or ^14^C-labelled NAD^+^ or ^14^C-labelled nicotinamide riboside [143,144], can also be translated to methods for the synthesis of the corresponding metabolites labelled with a stable isotope at a specific position, such as ^13^C-labelled NAD^+^ or ^13^C-labelled nicotinamide riboside [144]. In contrast to radioactive labels, working with stable isotope labels such as the biogenic isotopes ^2^H (deuterium), ^13^C, ^15^N, or ^18^O does not involve any health hazards and is not subject to regulations regarding radiation safety. The technology of isotope separation has enabled a continuous increase in the production of stable isotopes of light elements [145]. Compounds in which an atom like ^1^H, ^12^C, ^14^N, or ^16^O is replaced by a corresponding isotope with a higher atomic mass are of significant interest to numerous applications, because the chemical structure and physical properties remain unchanged. Major types of applications of labelling with stable isotopes, such as ^2^H, ^13^C, or ^15^N in stable bonds, in which the label is non-exchangeable under physiological conditions, are related to biological cells in both health and disease. These applications include quantification methods for specific metabolites, methods for analyzing metabolic fluxes and pathways, and the localization of metabolites through imaging methods. For quantification methods, which are important in diagnostics, stable isotope labelled metabolites, drugs, or metabolite like molecules are preferable. Alternatively, other approaches may be followed, like chemically labelling the unlabeled analytes using derivation reagents containing stable isotopes or employing quantitative NMR of native metabolites [146]. For stable isotope tracer methods, the analysis of carbon metabolic fluxes and pathways [147] and stable isotope resolved metabolomics [146]. This is important for analyzing disease-specific metabolic pathway alterations, such as cancer cell metabolism [148]. For these methods, as well as for imaging methods of biological and pathological processes [149,150], the ^13^C-labelled precursors or nutrients such as universally ^13^C-labelled D-glucose can be used for in vivo labelling. Metabolites labelled with an equal number of stable isotope atoms but at different positions can be distinguished using NMR as isotopomers. Metabolites which differ by their isotope number and composition can be distinguished through MS as isotopologues [146].

**Figure 10:**
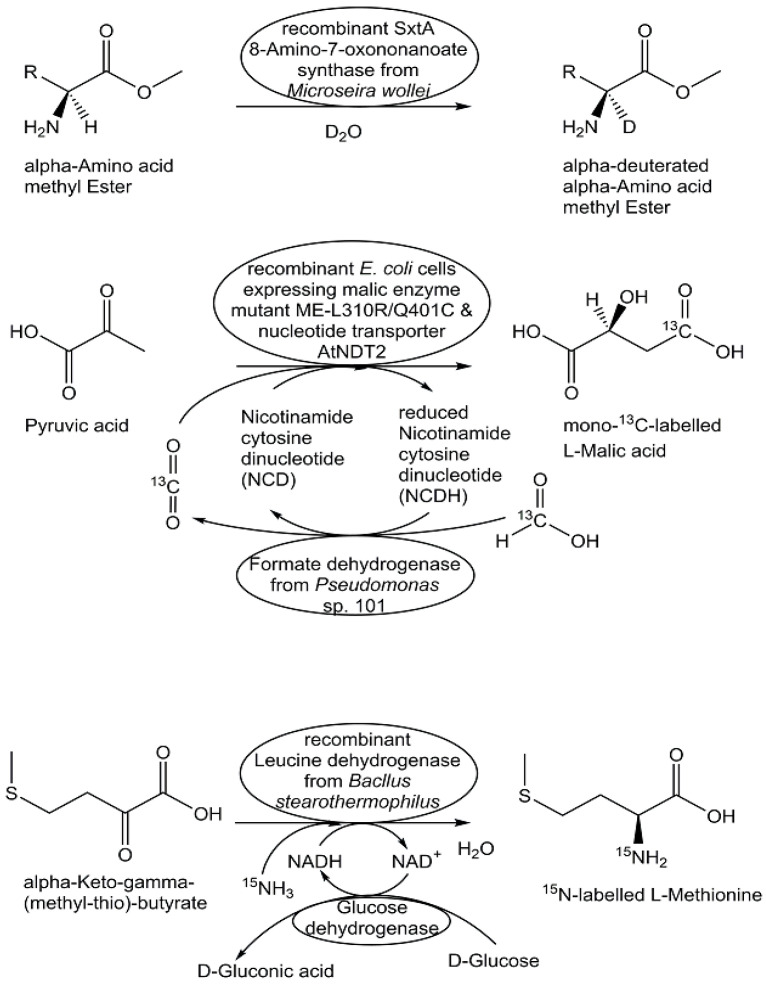
Selected biocatalytic systems for the synthesis of metabolites labelled with ^2^H-, ^13^C-, or ^15^N-stable isotopes.

The impressive advances of highly sensitive MS and NMR instrumentation, with their powerful methodologies and analyses with high information content, have shifted the interest to the use of the stable isotopes ^2^H, ^13^C, ^15^N, or ^18^O [151,152]. As isotope separation is demanding and requires highly specialized equipment and facilities for the production of stable isotope-labelled starting chemicals with high chemical and isotopic purity, the precious stable isotope-labelled starting materials should then be fully utilized for the synthesis of the desired metabolites. Therefore, highly selective synthetic methods are needed, which are able to efficiently incorporate to a high degree the stable isotope from the starting material into a defined position of the product and to completely convert the starting material to the target metabolite. The thermodynamics of biochemical reactions and the universe of biocatalysts provide a significant knowledge base from which suitable biocatalytic reactions for selective labelling with stable isotopes can be selected (see Figure 10).

Biocatalytic methods for site- and stereoselective deuteration are of much interest for short routes to deuterated metabolites, for example, in the synthesis of selectively deuterated phosphatidyl-*sn*-glycerol, amino acids deuterated in the α- and/or β-position, deuterated NAD^+^/ NADH cofactors, or deuterated aldehydes [153,154,155,156,157]. The biocatalytic synthesis of 3′,4′,5′,5′-tetradeuterated 5-phospho-D-ribosyl α-1-pyrophosphate (PRPP) was prepared from 3′,4′,5′,5′-tetradeuterated D-ribose through ribokinase-catalyzed phosphorylation and PRPP synthetase-catalyzed pyrophosphorylation [158]. The tetradeuterated PRPP was then converted through multistep enzymatic processes in high yields to the 3′,4′,5′,5′-tetradeuterated nucleotides ATP, CTP, GTP, and UTP [158].

The synthesis of ^13^C-labelled metabolites has been very useful for various metabolomics applications, such as for growing cells on media containing ^13^C-labelled carbon sources as nutrients [159], for detailed investigations of cellular metabolism and the functional properties of complex metabolic networks through ^13^C-based metabolic flux analysis [27,160], or for overcoming matrix effects in accurate and reliable metabolite analyses and quantitative metabolomics [161]. The key to these applications and the discovery of novel biosynthetic pathways like the deoxyxylulose phosphate pathway [162] has been the synthesis of ^13^C-labelled biochemicals, such as ^13^C-labelled acetate or isotope isomers (isotopomers) of D-glucose, where specific ^12^C-atoms are replaced by their ^13^C-isotopes. Enzymatic methods have facilitated access to ^13^C-labelled metabolites, such as monosaccharides [163], as well as ^13^C-labelled amino acids [164,165,166,167]. The use of very simple and inexpensive ^13^C-labelled precursors is thereby attractive, such as ^13^C-labelled pyruvate for the enzymatic synthesis of ^13^C-labelled aromatic amino acids [168]. Direct utilization of ^13^carbon dioxide is not only of significant interest for investigating the metabolism of photosynthetic organisms, but also for the biocatalytic synthesis of ^13^C-labelled biochemicals, such as ^13^C-labelled L-malate [169].

As many natural products contain nitrogen, the introduction of the stable nitrogen isotope ^15^N is very useful for discovering natural products and characterizing their biosynthetic pathways and metabolic intermediates [170]. Biocatalytic synthesis of ^15^N-labelled metabolites has been achieved in a straightforward way by introducing a stable isotope from ^15^NH_4_ salts, by using biocatalytic systems with isolated enzymes, or by making use of biosynthesis in whole cells growing in media containing ^15^NH_4_ salts. A very efficient NAD^+^-dependent amino acid dehydrogenase-catalyzed preparation has been demonstrated through the synthesis of ^15^N-labelled L-serine, L-methionine, and L-glutamic acid from the corresponding α-keto acids using alanine dehydrogenase, leucine dehydrogenase, and glutamate dehydrogenase, respectively, whereby NADH regeneration was performed using the glucose/glucose dehydrogenase system [171]. The four ^15^N-labelled cobalamin standards hydroxocobalamin, adenosylcobalamin, methylcobalamin, and cyanocobalamin have been prepared through biosynthesis by growing *Propionibacterium freudenreichii* whole cells in a chemically defined medium containing (^15^NH_4_)_2_SO_4_ instead of (^14^NH_4_)_2_SO_4_ [172].

In addition to using a single type of stable isotope for labelling, biocatalytic synthesis has also been attractive for the introduction of more than one type of stable isotope. Pentose phosphate and purine pathway enzymes, together with biocatalytic regeneration cycles for nucleoside triphosphate, folate, aspartate, glutamine, and NAD^+^, have been utilized for labelling purine nucleotides with ^13^C and ^15^N [29]. This flexible and robust one-pot biocatalytic system enabled the preparation of uniformly ^13^C- and/or ^15^N-labelled GTP, or ^13^C-labelled ATP in the C2- and C8-position from labelled serine, ammonium, glucose, and carbon dioxide [30].

## 5. Synthesis of Pharmaceutical Drug Metabolites

The development of new molecular entities for the effective treatment of human diseases with minimized side effects requires an understanding of its interactions with the biological cells of humans and their microbiome. The investigation of potential in vitro and in vivo pharmaceutical drug metabolism involving human and microbial enzymes, the biocatalytic reactions converting administered pharmaceutical drugs to derived metabolites, and the identity and biological activity of pharmaceutical drug metabolites, are of key importance to the treatment response, drug safety, and side effects. Knowledge about pharmaceutical drug metabolism reactions, data on drug metabolizing enzymes, and structures of drug metabolites has been growing significantly over the past years, as shown by their increasing numbers in the DrugBank database and its most recent version DrugBank 5.0 [173]. The complexity of pharmaceutical drug metabolism is increased further because pharmaceutical drugs not only interact with human metabolism but also with the human microbiome [174], as demonstrated by the biotransformation capabilities of the human gut microbiome towards numerous pharmaceutical drugs [175,176,177].

When applications of a pharmaceutical drug candidate show that it is enzymatically converted, from the site where it is administered to the desired drug action site, to a less active drug metabolite, this results in a poor treatment response and requires further drug development. For optimizing drug effectiveness while minimizing side effects, ensuring correct dosing, and avoiding drug overdoses of therapeutics with undesirable side effects, therapeutic drug monitoring has become an important tool for precision medicine, where isotope-labelled drugs are routinely used as reference standards for LC-MS/MS-analyses in clinical chemistry. Although pharmaceutical drug metabolism often leads to inactivation, there are also cases where therapeutic benefits may derive from biotransformation to a pharmacologically more active metabolite [178], for example, when a prodrug with better cell permeability is enzymatically converted to the active pharmaceutical in diseased cells. Pharmacologically inactive, or less active by three orders of magnitude, small molecular weight compounds, which are enzymatically converted in vivo to their active pharmaceuticals, have been developed through different paths. They have been discovered by chance, from rescuing a drug discovery project, or by designing a prodrug [179]. Chemically reactive or toxic drug metabolites have received increased attention, and the investigation of potential side effects, safety, and toxicity issues of pharmaceutical drug metabolites, which may potentially be formed through biotransformations in the human body, has also evolved with the “Metabolites In Safety Testing” (MIST) guidance and the framework for identifying, quantifying, and assessing human drug metabolite safety [180,181,182]. The investigations of the possible effects of such modified drugs require sufficient amounts of pure drug metabolites; therefore, straightforward methods for their synthetic access are highly desirable and can be highly significant for the timeline of projects. As selective chemical modification of complex drugs with stereocenters may be challenging [183], using biocatalysts which are involved in human drug metabolism provides an attractive selective approach [184].

**Figure 11:**
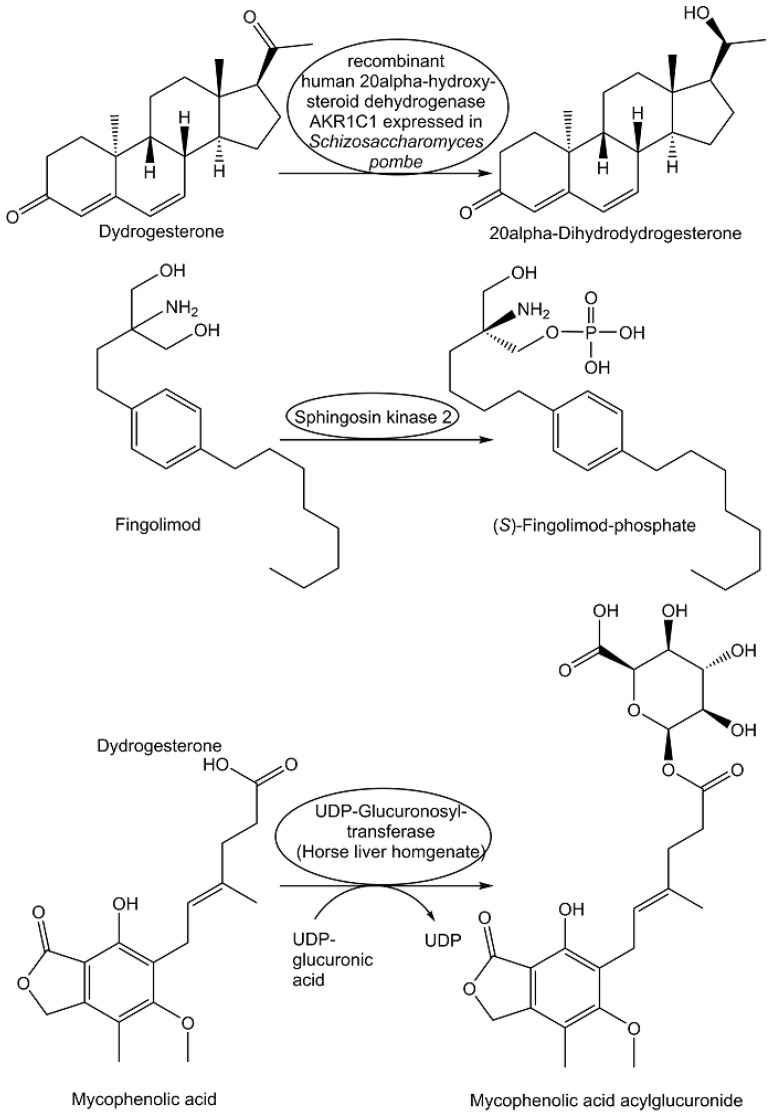
Selected biocatalytic systems for pharmaceutical drug metabolite synthesis.

When the orally active synthetic pharmaceutical drug dydrogesterone is used for treating progesterone deficiency and various gynecological conditions, human metabolism is responsible for the formation of the drug metabolite 20α-dihydrodydrogesterone. This drug metabolite has been prepared through the efficient stereo- and regioselective reduction of dydrogesterone (see Figure 11) catalyzed by recombinant human 20α-hydroxysteroid dehydrogenase AKR1C1 expressed in *Schizosaccharomyces pombe* [185]. With chemical reduction of the C20-keto group in dydrogesterone leading only to the 20β-dihydrodydrogesterone, biocatalytic reduction is key for obtaining 20α-dihydrodydrogesterone, which is also pharmacologically active [185]. The drug metabolite (*S*)-fingolimod-phosphate, which is a modulator of sphingosine 1-phosphate receptor 1, is formed in vivo through sphingosine kinase 2-catalyzed phosphorylation (see Figure 6) of the drug fingolimod, which has been approved as a pharmaceutical drug for the therapy of multiple sclerosis in more than 80 countries [186]. As different forms of mycophenolic acid have various therapeutic applications as pharmaceutical drugs, for example, as immunosuppressants, their pharmacologically active metabolite, mycophenolic acid acylglucuronide has attracted interest, as it also inhibits inosine monophosphate dehydrogenase II like mycophenolic acid [187]. For the biocatalytic synthesis of the acylglucuronide of mycophenolic acid, only horse liver homogenate was found to catalyze the glucuronidation of mycophenolic acid, using UDP-glucuronic acid as donor, but the acylglucuronide was formed in a 1:1 mixture with the 7-*O*-glucuronide [187]. Through the optimization of the reaction temperature, the concentrations of the liver homogenate, and the UDP-glucuronide, the degree of conversion was increased to 54% and an acylglucuronide to 7-*O*-glucuronide ratio of 4.9:1 could be obtained, leading to the drug metabolite mycophenolic acid acylglucuronide (see Figure 11) in a >95% purity and a 34% isolated yield [187].

Biocatalytic transformations of synthetic pharmaceutical drug derivatives through human metabolism in vivo are important aspects in the design of prodrugs in order to overcome barriers in delivering and releasing the parent drug, and to improve cell permeability and water solubility [188].

Although many drug metabolites are pharmacologically inactive or much less active than their parent drugs, as exemplified by the pharmacologically inactive 7-*O*-glucuronide of mycophenolic acid as the major mycophenolic acid metabolite in humans, ensuring synthetic access is important [189]. The 7-*O*-glucuronide of mycophenolic was obtained with 97% purity through biocatalytic glucuronidation of mycophenolic acid using horse liver homogenate as the biocatalyst and UDP-glucuronic acid as the donor [187].

Chemically reactive drug metabolites, which are formed by human metabolism, can react with relevant molecules of biological cells and lead to functional changes and adverse drug reactions [190]. In the drug metabolism of the nonsteroid anti-inflammatory drug diclofenac, which is used for treating rheumatoid disorders, the two metabolic pathways of the cytochrome P450-catalyzed oxidation to the corresponding chemically reactive quinone imine metabolites and the enzymatic glucuronidation are thought to be involved in several adverse drug reactions [191,192]. Cytochrome P450 enzymes have been shown to catalyze the drug conversion to chemically reactive metabolites, which can cause toxic effects, such as the oxidation of acetaminophen to the toxic metabolite *N*-acetyl-*p*-benzoquinone imine catalyzed by human cytochrome P450 2E1, 1A2, and 3A4 [192,193,194].

## 6. Synthesis of Metabolite-like Compounds

The concepts of natural product-likeness [195,196] and metabolite-likeness [197,198] are attractive because the transport of natural products, nutrients, and metabolites is omnipresent in biological organisms. Natural-product-like and metabolite-like structures have been found to be present in a significant number of approved pharmaceutical drugs [99,199].

**Figure 12:**
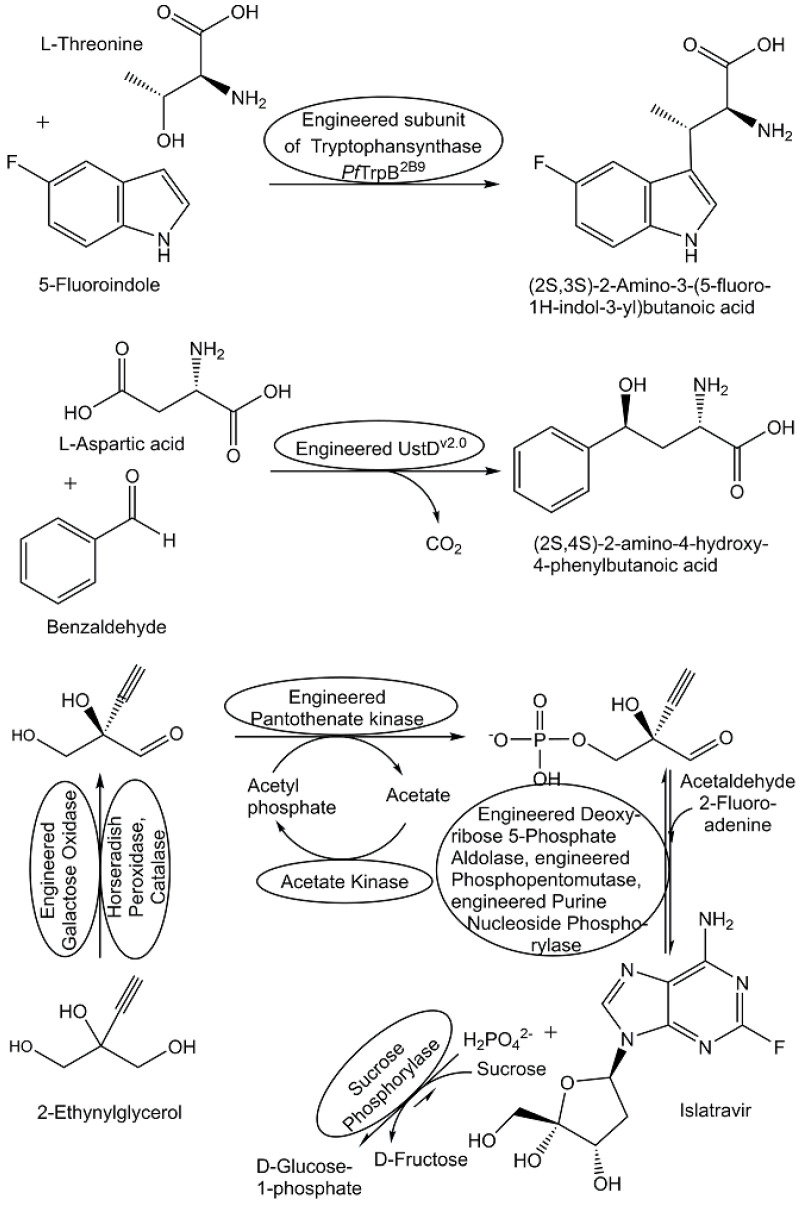
Biocatalytic synthesis of selected metabolite-like compounds.

Variations in metabolite and natural product structures have attracted increasing interest due to the functional group differences which have been found to exist between natural products and synthetic molecules [200], the changing structural characteristics and properties of approved pharmaceutical drugs over time [201,202], and the interactions between metabolites and natural products with target proteins, including biosynthetic and transport proteins [203]. With the increasing knowledge about specific interactions between metabolites of the human microbiome and human disease-relevant biomolecules such as ligand–receptor or enzyme inhibitor–enzyme interactions, the concept of utilizing microbial metabolites as rich molecular spaces is very attractive for discovering metabolite-like compounds as novel pharmaceutical drugs for highly precise therapies and the derisking of adverse drug reactions [204,205]. The development of metabolite-like compounds from bacterial tryptophan metabolism for discovering novel ligands which bind directly to the pregnane X receptor and are not cytotoxic is of much interest for further development and opens up a wide range of opportunities [206]. Excellent biocatalytic tools and methodologies have been developed for synthesizing non-natural chiral amino acids with high enantioselectivity, and the diversity of possible functional groups and chiral centers in non-natural amino acids is attractive for their use as fragments in small molecule pharmaceutical drugs (for some examples, see Figure 12) for the therapy of diseases [207,208,209,210].

Biocatalytic reactions for highly stereoselective formation of carbon–carbon bonds is of central importance for the synthesis of both natural metabolites as well as metabolite-like compounds (see Figure 12), but practical applications are not very common [208]. The thermodynamic advantage of decarboxylative carbon–carbon bond formation, as discussed previously in the context of transketolase-catalyzed reactions, has been demonstrated by reactions catalyzed by the enzyme UstD and its evolved variants [210]. This leads to the stereoselective formation of a series of γ-hydroxy-α-amino acids from various aldehydes and L-aspartic acid with the release of carbon dioxide (see example in the middle of Figure 12). The thermodynamic equilibrium of a stereoselective aldol reaction catalyzed by an engineered deoxyribose 5-phosphate aldolase has been overcome by coupling it to the subsequent reactions in the biocatalytic synthetic route to islatravir (see example at the bottom of Figure 12) and removing the inorganic phosphate side product [211]. Numerous advances have been achieved in recent years in stereoselective alkylations, acylations, oxidative carbon–carbon coupling reactions, cyclizations, and carbene transfer reactions [212]. The synthesis of various pyrroloindolines has been achieved through the regio- and stereoselective methylation of various indoles at their C3-position when catalyzed by the *S*-adenosyl-L-methionine (SAM)-dependent methyltransferase PsmD from *Streptomyces griseofuscus*, whereby SAM was regenerated by methyliodide and halide methyltransferase from *Chloracidobacterium thermophilum* [213]. Recombinant *trans*-α-hydroxybenzylidenepyruvate hydratase-aldolase (NahE) has been demonstrated to catalyze stereoselective Michael addition reactions of pyruvate to various β-nitrostyrenes, from which then the corresponding β-aryl-γ-nitrobutyric acids can be obtained [214].

The advances in the characterization and engineering of enzymes catalyzing routine reactions in organic chemistry laboratories, such as Diels–Alder reactions, Claisen, and Cope rearrangements are very promising for translating biocatalytic complexity-generating reactions into routine operations [215]. The development of highly active Diels-Alderases from nature, through engineering, and by design for catalyzing highly stereoselective intermolecular [4+2] cycloaddition reactions is of great interest for the synthesis of natural products [216,217] and for catalyzing abiological hetero-Diels–Alder reactions [218]. Their versatility has been increased by reversing the *exo*-selectivity of natural Diels-Alderases to the endo-selectivity of an engineered Diels-Alderase, catalyzing the intermolecular [4+2] cycloaddition of the same substrates with high enantioselectivity and broadening the scope of the dienes and dienophiles accepted as substrates [219].

Combining biocatalytic and chemical reaction steps in chemoenzymatic syntheses offers new opportunities for overcoming challenges and diversifying metabolite structures. Advances have been achieved in the synthesis of diversified compounds of the plant metabolite *cis*-(+)-12-oxophytodienoic acid [220], a variety of non-natural nucleosides and nucleoside building blocks (see Figure 12), as well as in the synthesis of nucleoside analogue drugs through enzymatic cascades [221,222].

## 7. Discussion

The growing number of metabolite structures identified in biological organisms and the renewed interest in the elucidation of the fundamental roles and useful properties of metabolites have also increased the necessity of developing analytical and synthetic methods and tools for a great diversity of metabolites. The identification of natural metabolic pathways has provided great starting points for the development of straightforward biocatalytic synthesis routes inspired by nature. Biocatalytic systems offer particular benefits not only for producing naturally occurring metabolites but also for stable isotope-labelled metabolites, drug metabolites, and non-natural metabolite-like compounds. The molecular economy and reduced complexity of highly selective and protecting group-free biocatalytic reactions have enabled resource-efficient and robust production procedures for these metabolite classes. When the extraction yields of naturally occurring metabolites from biological resources are rather low and quite variable, or if challenging and lengthy chemical routes using protection–deprotection schemes require significant purification steps, biocatalytic production procedures using recombinant biocatalysts are a preferred choice. The strategic advantages of biocatalytic systems enable the fast generation of molecular complexity through shorter synthetic routes in a straightforward way, as demonstrated by the development of the biocatalytic synthesis of the antiviral compound islatravir. The cascade of the three biocatalytic reaction steps leading to a single stereoisomer of islatravir from achiral 2-ethinylglycerol (see Figure 12) shortens the number of reaction steps to less than half of the reaction steps reported in previous routes [211].

Engineering enzymes towards desired activities and selectivities under certain reaction conditions and the combination of the necessary enzymes in a cascade reaction enable us to overcome unfavorable thermodynamic equilibria, enzyme inhibition, and the isolation of intermediates. Minimizing the time for the development, manufacturing, and supply of a compound in high demand and urgently needed is always very critical, but it has been even more crucial during the COVID-19 pandemic. The fast development of a short scalable biocatalytic route to the antiviral agent Molnupiravir using engineered ribosyl-1-kinase and uridine phosphorylase as key enzymes was impressive [223]. The advantages of utilizing a biocatalytic reaction cascade for preparing complex non-natural small molecules in one pot have also been demonstrated through the efficient biocatalytic synthesis of the cyclic dinucleotide MK-1454 using three engineered kinases and an engineered cyclic guanosine–adenosine synthase as key enzymes [224]. An isolated yield of 62%, based on the starting material of the nucleotide monothiophosphates, has been achieved for a single diastereomer of MK-1454 without the use of any protecting group [224]. When manufacturing at larger scale is needed due to increasing demand, raw materials, resource efficiency, and reliability issues have become increasingly important. Sustainability benefits can also be achieved when biological resources which are rare or in danger of becoming extinct are preserved and abundant bio-based resources are used for metabolite production using biocatalytic systems.

Excellent opportunities appear for exploring uncharted territory regarding novel metabolites and their biological functions [225], natural product drugs [226,227], and metabolite-like compounds. Discovering and characterizing novel enzyme functions and pathways from nature, as well as engineering and evolving enzymes which catalyze reactions that are new to nature [228,229], are important for extending the frontiers of biocatalytic reactions in syntheses. Unlocking the power of enzymes can transform the synthesis of metabolites, natural products, and non-natural small molecules derived thereof in various ways, from individual enzymatic reactions in chemoenzymatic synthesis to their full utilization in enzymatic total synthesis [230,231,232].

## 8. Future Directions

With the rising gap between the number of known metabolites and the number of metabolites whose syntheses have been reported, there are more than enough concrete problems to be solved, such as the synthesis of important metabolites with high biological activity and inherent instability, or biologically active metabolites which are synthesized by bifunctional enzymes carrying synthetic and degrading functions within the same protein. Rapid advances in enzyme engineering and computational methodologies have been outlined and appear promising on the road towards the design of robust enzymes with desired enzyme functional properties [233]. Many roads can lead to discovering novel enzymes from nature, such as guiding the correct assignment of enzyme functions to gene annotations and domains of unknown functions on genomes [234,235,236], unlocking natural product biosynthetic enzymes from metagenomes [237], or identifying missing enzymes in biosynthetic pathways to metabolites [238]. Mining microbial genomes for biosynthetic gene clusters [239] and deciphering precise genome–metabolome relationships of bacteria and fungi are very promising approaches for finding novel biosynthetic enzymes and pathways to novel metabolites [240,241,242,243]. Advanced metabolic engineering and synthetic biology tools and methodologies [244] and efficient gene expression to highly functional and fit-for-use enzymes are key for engineering novel biocatalytic pathways in viable and sustainable production processes. Therefore, the future of synthesizing naturally occurring metabolites [245,246], metabolite-like compounds, drug metabolites, and stable isotope-labelled metabolites using biocatalytic systems looks bright.

**Table 2:** Overview of Natural Metabolites covered in this review.

Metabolites	Metabolic Pathway	Reference
Acetic acid	Carbon metabolism	[58]
(-)-Ambrox	Homofarnesol cyclization	[108]
4-Androstene-3,17-dione	Steroid biosynthesis	[90]
L-Argininosuccinate	Urea cycle	[129,130]
Artemisinin	Artemisinin bioynthesis	[103]
Avermectin	Polyketide biosynthesis	[102]
Azadirachtin	Tetranortriterpenoid biosynthesis	[61]
Cholesterol	Steroid biosynthesis	[67,69]
Citric acid	Citrate cycle (TCA cycle)	[81]
1α,25-Dihydroxyvitamin D2	Steroid biosynthesis	[137]
1α,25-Dihydroxyvitamin D3	Steroid biosynthesis	[135]
24*R*,25-Dihydroxyvitamin D2	Steroid biosynthesis	[138]
24*R*,25-Dihydroxyvitamin D3	Steroid biosynthesis	[136]
17β-Estradiol	Steroid biosynthesis	[89]
L-Glyceraldehyde	Pentose and glucuronate interconversions	[115,116]
D-Glyceraldehyde-3-phosphate	Embden-Meyerhof-Parnas pathway Carbon fixation in photosynthesis Pentose phosphate pathway	[117]
L-Glyceraldehyde-3-phosphate	Isomerase bypass	[113,114]
D-Glycerate-2-phosphate	Embden-Meyerhof-Parnas pathway	[118]
25-Hydroxyvitamin D2	Steroid biosynthesis	[133]
25-Hydroxyvitamin D3	Steroid biosynthesis	[131,132,133,134]
2-Keto-3-deoxy-D-galactonate	Galactose metabolism	[124]
2-Keto-3-deoxy-D-gluconate	Non-phosphorylative Entner-Doudoroff pathway Pentose phosphate pathway	[125]
2-Keto-3-deoxy-6-phosphogluconate	Entner-Doudoroff pathway Pentose phosphate pathway	[123]
2-Keto-3-deoxy-D-xylonate	Pentose and glucuronate interconversions	[124]
Palytoxin	Palytoxin biosynthesis	[74]
Penicillin V	Penicillin biosynthesis	[59]
Nω-Phospho-L-arginine	Phosphagen pathway	[126]
Pyridoxamine-5’-phosphate	Vitamin B6 metabolism	[128]
Shikimic acid-3-phosphate	Shikimate pathway	[127]
D-Tagatose-1,6-diphosphate	Galactose metabolism D-Tagatose pathway	[122]
Taxol	Taxol biosynthesis	[63,100,101]
Testosterone	Steroid biosynthesis	[91]
Urea	Urea cycle	[57,79,80]
Vinblastine	Indole alkaloid biosynthesis	[75,238]
Vitamin B12	Cobalamine biosynthesis	[70,71,72,73,107]
D-Xylulose-5-phosphate	Pentose phosphate pathway	[119,120,121]
L-Xylulose-5-phosphate	Pentose and glucuronate interconversions	[121]

## Figures and Tables

**Table 1:** Selection of Species-specific Metabolite Databases.

Biological Species	Name of Species- Specific Metabolite Database	Abbreviation of Metabolite Database Name	Website of Metabolite Database	Reference	Accessed Date for the URL
Human	Human Metabolome Database	HMDB	https://hmdb.ca/	[14]	accessed on 16 July 2023
Human Microbiome	Human Microbial Metabolome Database i	MiMeDB	https://mimedb.org/	[15]	accessed on 16 July 2023
*Escherichia coli*	*Escherichia coli* Metabolome Database	ECMDB	http://www.ecmdb.ca/	[16]	accessed on 16 July 2023
*Pseudomonas* *aeruginosa*	*Pseudomonas aeruginosa* Metabolome Database	PAMDB	http://pseudomonas.umaryland.edu/	[17]	accessed on 16 July 2023
*Streptomyces* sp.	*Streptomyces* Natural Products Database	StreptomeDB	http://www.pharmbioinf.uni-freiburg.de/streptomedb/	[18]	accessed on 30 July 2023
Cyanobacteria	Comprehensive database of secondary metabolites from cyanobacteria	CyanoMetDB	https://zenodo.org/record/7922070/	[19]	accessed on 30 July 2023
Myxobacteria	Myxobacterial Natural Product Database	MyxoDB	https://www.myxonpdb.sdu.edu.cn/	[20]	accessed on 30 July 2023
Yeast	Yeast Metabolome Database	YMDB	http://www.ymdb.ca/	[21]	accessed on 16 July 2023
Bovine	Bovine Metabolome Database	BMDB	https://bovinedb.ca/	[22]	accessed on 16 July 2023
Tomato	Tomato Metabolome Database	TOMATOMET	http://metabolites.in/tomato-fruits/	[23]	accessed on 24 July 2023
